# Aquatic Contamination in Lugano Lake (Lugano Lake Ecological Reserve, Buenos Aires, Argentina) Cause Negative Effects on the Reproduction and Juvenile Survival of the Native Gastropod *Biomphalaria straminea*

**DOI:** 10.3389/fphys.2022.954868

**Published:** 2022-07-14

**Authors:** María Gimena Paredes, Karina Alesia Bianco, Renata J. Menéndez-Helman, Gisela Kristoff

**Affiliations:** ^1^ Laboratorio de Evaluación Ecotoxicológica del Agua: Invertebrados Nativos y Otros Modelos, Departamento de Química Biológica, Facultad de Ciencias Exactas y Naturales, Instituto de Química Biológica de la Facultad de Ciencias Exactas y Naturales (IQUIBICEN)-CONICET, Universidad de Buenos Aires, Buenos Aires, Argentina; ^2^ Laboratorio de Enzimología, Estrés Oxidativo y Metabolismo, Departamento de Química Biológica, Facultad de Ciencias Exactas y Naturales, Instituto de Química Biológica de la Facultad de Ciencias Exactas y Naturales (IQUIBICEN)-CONICET, Universidad de Buenos Aires, Buenos Aires, Argentina

**Keywords:** biomonitoring, development, hatching, invertebrates, offspring survival, Matanza-Riachuelo basin, snails, water pollution

## Abstract

Lugano Lake is located in an Ecological Reserve of Buenos Aires City. Biomonitoring of its water quality is essential due to its importance as a place for recreation and protection of native species. *Biomphalaria straminea* is a native hermaphrodite aquatic gastropod that inhabits different freshwater bodies of Argentina and was recently selected as a potential bioindicator. We propose this study as a first approach to assessing specific organisms’ use in biomonitoring of urban wild reserves, and the usefulness of reproduction assays. *B. straminea* survival, behavior, reproduction success and offspring survival after the exposure to water samples from Lugano Lake (L1, L2, and L3) were evaluated. Temperature, pH, conductivity and dissolved oxygen were registered *in situ*. Samples were transported to the laboratory and chemical analysis and bioassays were performed using 20 snails per site. A control group with tap water was added. Egg masses were separated, exposed individually and observed daily using a stereoscopic microscope. After hatching, juveniles were placed in tap water and offspring survival was registered at the first, second, third and fourth months after the beginning of the assay. High levels of conductivity, turbidity and nutrients were obtained. Ammonium and nitrite were higher than the guideline level for the protection of aquatic life. During the bioassay 20% of the snails (L2 and L3) showed abnormally protruding of the head-food region. The number of eggs and embryonated eggs per mass did not differ between treatments. Egg masses exposed to water samples from the lake presented overlapping and abnormal eggs and arrested embryos. Besides, low % of hatching (L1: 33%, L2: 42%, and L3: 16%) and juvenile survival after the first (L1:14%; L2:78%) and second month (L1: 60%) were noted. In the control group, 85% of hatching and 100%–90% of survival were observed. Our results suggests the presence of pollutant in the lake. *B. straminea* seems to be a sensitive local species. *Biomphalaria* spp. reproduction assays can provide a valuable endpoint for toxicity and risk assessments and a usefulness tool for biomonitoring water quality.

## Introduction

Urban ecological reserves are essential areas, especially in densely populated cities, in order to protect the environment and native species, generate spaces for recreational and educational purposes and therefore improve life’ s quality. Water contamination due to human activity generates negative impacts in these areas so water monitoring should be a priority in environmental management.

Different measurable water quality variables can be used to evaluate or predict the condition or pollution degree of water bodies (USEPA 2000, 2002). Dissolved oxygen (DO), pH, nutrients (ammonium ions (NH_4_^+^), nitrate ions (NO_3_^−^) or phosphate ions (PO_4_^3−^) concentrations), chemical oxygen demand, electrical conductivity are parameters that are useful as indicators of water quality. There are numerous water quality indices (WQI) from National and International Agencies, which consider these physicochemical parameters for water assessment and pollution control. Nevertheless, the use of physicochemical parameters alone does not provide enough information about the biodisponibility of pollutants and the damage or possible damage caused to species; therefore, it is advisable to complement them by using biological indicators (Burgess et al., 2013).

Freshwater gastropods have been suggested as useful model organisms to evaluate the toxicity of chemicals compounds and environmental biomonitoring (Tripathi and Singh, 2004; Rivadeneira et al., 2013; Tallarico et al., 2014; Otero and Kristoff, 2016; Agrelo et al., 2019; Herbert et al., 2021; Siqueira et al., 2021; Stein et al., 2021). Their slow mobility and difficulty to detoxify pollutants make them sensitive species for water contamination (Gao et al., 2017). Generally, embryos and juveniles are more sensitive to pollutants than adult gastropods (Leung et al., 2007; Khangarot and Das, 2010; Attia et al., 2017; Agrelo et al., 2019), so toxicity data using the most vulnerable life stage would offer protection for all developmental stages in the environment (Caixeta et al., 2022). However, not all gastropods are useful for reproduction assays due to their low rate of reproduction and slow development and growth, such as *Chilina* spp. (Gastropoda, Chilinidae) (Cossi et al., 2017).

*Biomphalaria* spp. (Gastropoda, Planorbidae) has many advantages that make it a suitable model system in reproduction assays such as high egg production, fast development and growth, short life cycles and transparent layer covering the embryos (Cossi et al., 2020; Caixeta et al., 2022). These snails are simultaneous hermaphrodites, and are able to reproduce by both self-fertilization and cross-fertilization. Snails lay their eggs in a gelatinous capsule or egg mass (Figure 2) where embryos develop and juveniles hatch after a few days (Yipp, 1983). Different parameters such as the number of egg masses laid, eggs and embryonated eggs per mass, development delays, morphological changes, time and success of hatching, offspring survival and malformations have been evaluated during toxicity tests (Kristoff et al., 2011; Silva et al., 2018; Caixeta et al., 2021).

*Biomphalaria straminea* is a native species with a wide distribution in Argentina (Rumi et al., 2008, 2006). Due to they are easily maintained in laboratory cultures, have a great reproductive potential, rapid embryonic development and growth, early sexual maturation and small size, this species is a useful model to perform toxicity test. In previous studies, we have reported that juveniles hatch after 6–8 days in laboratory conditions (23 ± 1°C; 12:12 h Light:Dark) (Cossi et al., 2018; 2020). Taking into account these features and due to its sensitivity to pesticides (Bianco et al., 2014; Cossi et al., 2018, 2020), it was selected as a potential bioindicator of environmental health in Argentina (Informe del Estado del Ambiente, 2017).

**FIGURE 1:**
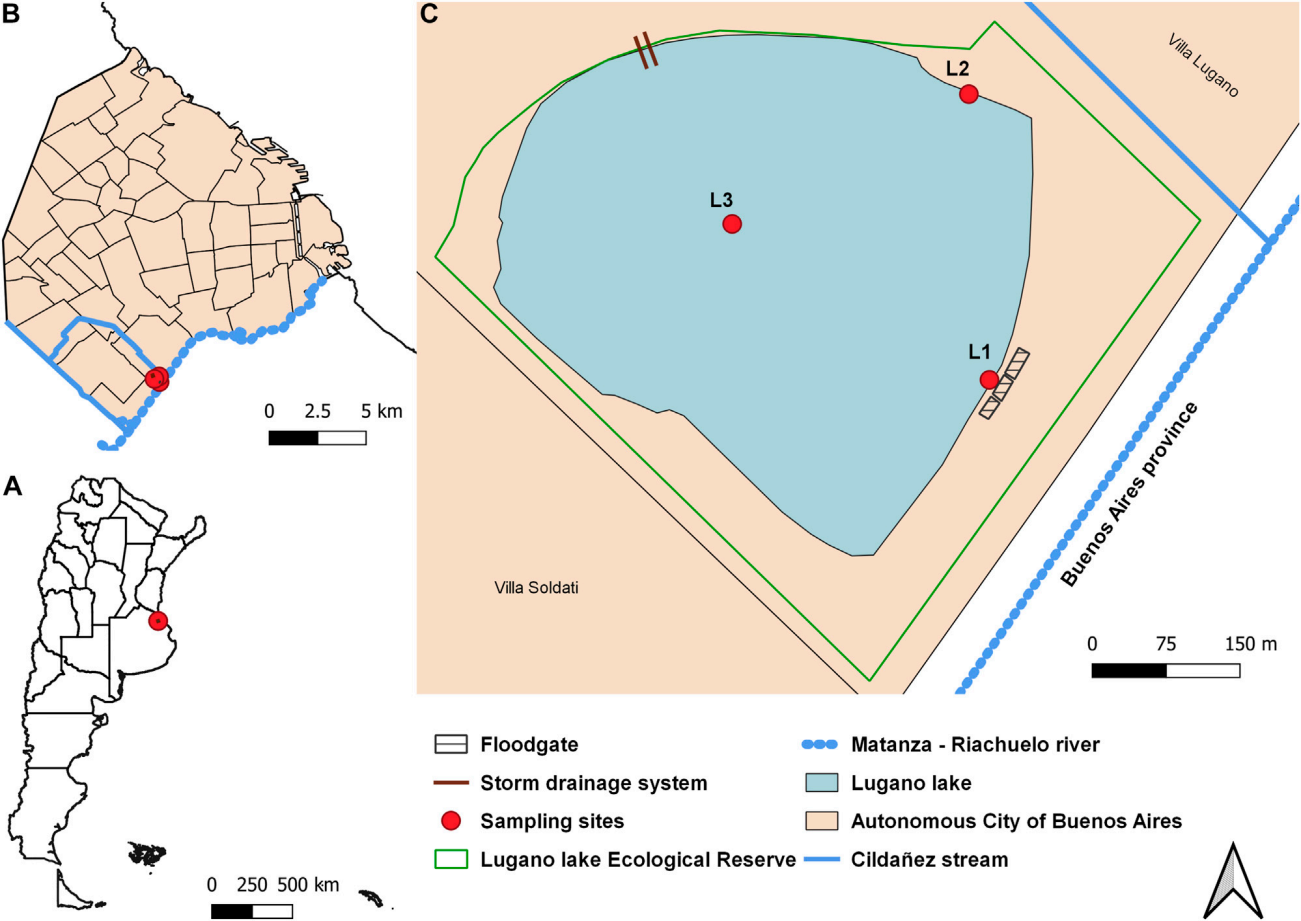
Geographical location. **(A)**: Argentina, **(B)**: Buenos Aires City, **(C)**: Lugano Lake inside of the Ecological Reserve and samples sites (L1, L2, and L3).

Lugano Lake is an urban artificial lake located in the Lugano Lake Ecological Reserve, Buenos Aires City, Argentina (Figure 1). The reserve has 36 hectares that surround the lake and it is one of the three Ecological Reserves of the city. The lake has 550 m in diameter and the main depth is 1.5 m. This artificial lake was built in 1940 to act as a relief for the Cildañez Stream, avoiding the overflows of the Riachuelo River and therefore the floods over Buenos Aires City (Lopez Sardi and Larroudé, 2020). The Cildañez Stream and the Riachuelo River are part of the Matanza-Riachuelo Basin. This basin presents a significant degree of contamination, especially due to industrial and domestic waste. Low values of dissolved oxygen (DO) and high values of nutrients and certain metals such as lead and copper have been indicated by water quality reports of Water Matanza-Riachuelo Basin Authority (ACUMAR (Autoridad de Cuenca Matanza Riachuelo), 2018). Lugano Lake receives discharges from the Riachuelo, the Cildañez and from nearby neighborhoods, especially in times of rain and floods. The presence and abundance of species that inhabit it are useful tools as indicators of environmental health. Different vertebrates such as birds, fish and turtles have been reported in the reserve, however there is no data on the species of invertebrates that inhabit it.

We propose this study as a first approach to assessing specific organisms’ use in biomonitoring of urban wild reserves, and the usefulness of reproduction assays for water biomonitoring. Adult snails’ survival and behavior, embryos’ development, hatching success, and offspring survival in *B. straminea* exposed to different water samples from Lugano Lake (Lugano Lake Ecological Reserve) were evaluated.

## Materials and Methods

### Organisms

The original population of *B. straminea* snails were collected from an urban lake (Regatas Lake, Buenos Aires City) in 2014 and characterized as *B. straminea* (Bianco et al., 2012). Then, snails’ population were reared in our laboratory until now in aerated glass aquaria (7–16 L) with dechlorinated passive tap water (TW) at a temperature of 23 ± 1°C and under a photoperiod 12:12 h (Light:Dark). The snails are fed *ad libitum* with *Lactuca sativa* var. capitata L. (butterhead lettuce) previously washed with TW and cut.

### Water Sampling

Water sampling was carried out (spring 2021) from 3 different sites of Lugano Lake (Figure 1). L1: 34°40′54.5″S–58°26′35.1″W; L2: 34°40′44.8″S–58°26′37.0″W and L3: 34°40′49.1″S–58°26′45.9″W. Water was taken from the surface using a 10 L plastic bucket with a rope (L1: from the concrete wall that delimits the lake and it is located near the gates that connect it with the Riachuelo River, L2: from the shore; and L3: from a boat). Water samples were collected in polyethylene plastic containers (two 5-L containers for the bioassays and one 1-L bottle for the physicochemical determinations). Each container was rinsed with the water from the same place before taking the samples.

Water samples were immediately transported to the laboratory located in the Faculty of Exact and Natural Sciences, University of Buenos Aires on ice packs.

### Physicochemical Parameters

Physicochemical parameters such as pH, conductivity, and temperature were measured *in situ* using a Hanna HI 9811-5 multiparameter meter, whereas DO was determined using a Hanna HI 9145 portable oximeter. Both instruments were previously calibrated and used according to the manufacturer’s instructions.

An aliquot (250 ml) of each water sample was filtered through cellulose acetate membranes of 0.45 µm pore diameter to carry out the chemical analyses. Standard water analysis techniques (APHA, 1998) were performed with a Hach DR/2010 spectrophotometer and Hach kits (Hach CO., Loveland, CO, United States) for nitrate, nitrite, ammonium, and phosphate determinations. Alkalinity and total hardness were monitored using Hach test Kits: Model AL-DT (Digital Titrator/ Sulfuric Acid method) and Model HAC-DT (Digital Titrator/EDTA method), respectively. All the parameters were also measured in TW.

### Bioassays: Experimental Design

Adult snails of similar weights (0.08 ± 0.008 g) were selected to carry out the bioassays. Snails were left 1 week in glass containers (1 L) for acclimation and depuration to ensure they released any egg mass they could have been carrying from the aquaria. 20 adult snails were exposed to each unfiltered water sample (750 ml) (L1, L2, L3) in glass containers (1 L) for 7 days. Another 20 snails were placed in containers with TW. Bioassays were performed under controlled conditions of temperature (23 ± 1°C) and photoperiod 12:12 h (Light:Dark). During this time, adult organisms were fed *ad libitum* with *L. sativa* var. capitata L. (butterhead lettuce). During the bioassay, mortality, neurotoxicity signs and behavior alterations were registered.

### Lethality

Organisms were considered dead if no movement occurred in response to mechanical stimuli, if they remained permanently retracted inside their shells, or if shells were empty (Cossi et al., 2018). The number of dead snails was registered and % of lethality was calculated on total snails per treatment.

### Neurotoxicity Signs and Behavior Alterations

Behavioral alterations and neurotoxicity signs were evaluated visually and included those alterations previously observed in freshwater gastropods such as weak or lack of adherence, abnormally protruded head-foot region, decrease in spontaneous movements and escape from solutions (Tejedor and Kristoff, 2020; Herbert et al., 2021). The number of snails with some alteration was registered and the percentage was calculated on the total snails per treatment.

### Reproduction Parameters

At day 7 of the bioassay, the number of laid egg masses per treatment was recorded. Masses were carefully removed from the containers and were placed individually in plaques of 6-well plates (10 ml) containing 8 ml of each water sample (L1, L2, L3 or TW). Broken masses were discarded. Bioassay was carried out under controlled conditions of temperature (23 ± 1°C) and photoperiod 12:12 h (Light:Dark). Total egg masses (46) were examined daily under a stereoscopic microscope (Nikon SMZ645). The number of eggs per mass, embryonated eggs per mass, morphological abnormalities and arrested eggs were registered. The hatching success was determined by counting the number of hatched juveniles per egg mass. The percentage of hatching was calculated on the total number of eggs and on the total number of embryonated eggs.

### Survival at the First Month

After hatching, the water in each well was replaced by TW (8 ml) and juveniles were fed with lettuce. TW was renewed once a week. Juvenile survival was evaluated daily using a stereoscopic microscope. Juveniles were considered dead when they showed lack of mobility, discoloration, visceral mass exposure and lack of heartbeats. After 1 month, alive juveniles were counted. The percentage of juvenile survival at the first month was calculated on the number of hatched juveniles per treatment.

### Juvenile Survival After the First Month

After the first month, 10 snails per treatment (randomly assigned) were placed in 250 ml glass containers (due to the growth of the juveniles) with TW and the number of alive snails were counted monthly (second, third and fourth month). Each month, the percentage of survival was calculated on the total of snails per treatment (10). During this time, TW was renewed once a week.

### Statistical Analysis

Assumptions of normality (Shapiro Wilk test) and homogeneity of variances (Bartlett test) were verified.

Significant differences between groups of the number of eggs and the number of embryonated eggs per mass were analyzed using a one-way ANOVA. The percentage of adult survival, behavior alterations and hatching and offspring survival were analyzed using Fisher test and Chi-squared test. All statistical tests were performed using 0.05 as the level of significance. The package R was used for all statistical analysis.

## Results

### Water Characteristics and Physicochemical Parameters

Inside the Lugano Lake Ecological Reserve, the presence of turtles, different species of birds and ducks as well as native vegetation and trees, were observed. Water in the edges looked green due to the presence of algae, mainly in site L1 (near the mouth of the Cildañez stream in the Riachuelo), while there was less turbidity in the center of the lake. High densities of pelagic algae could decrease water transparency and increase turbidity. In L2 the presence of vegetation, both aquatic and terrestrial, and freshwater invertebrate shells including *Biomphalaria* spp. were observed.

Table 1 summarized the physicochemical parameters measured *in situ* in the studied area (conductivity, pH, temperature and DO). In the laboratory, these parameters were also measured in TW being the conductivity 270 μS cm^−1^, pH 7.1, temperature 22.6°C and DO 8.98 ppm and 96.6%.

**TABLE 1:** Physicochemical parameters obtained *in situ* in water from sites L1, L2, and L3, Lugano Lake, Buenos Aires.

Water Samples	Conductivity	pH	Temperature	DO	
	µS.cm^−1^	—	°C	ppm	%
L1	1,480	8.5	23.4	12.36	137.0
L2	1,440	8.5	28.0	11.16	130.3
L3	1,370	8.4	26.7	9.50	112.7

Table 2 summarized physicochemical parameters measured in the laboratory in water from L1, L2, and L3 and in TW.

**TABLE 2:** Physicochemical parameters measured, in the laboratory, in tap water (TW) and water from sites L1, L2, and L3, Lugano Lake, Buenos Aires. LD: limit of detection.

Water samples	Alkalinity	Ammonium (NH_4_^+^)	Nitrites (NO_2_^−^)	Nitrates (NO_3_^−^)	Hardness	Turbidity	Phosphates
	mg CaCO_3_.L^−1^	mg.L^−1^	mg.L^−1^	mg.L^−1^	mg CaCO_3_ L^−1^	NTU	mg.L^−1^
TW	55	0.01	0.016	4.43	44	1.0	0.16
L1	328	4.29	0.046	<LD	208	125.0	3.80
L2	302	3.35	1.123	4.87	208	82.5	4.00
L3	316	5.02	0.046	<LD	204	75.3	1.60

### Lethality

During the bioassay, only one snail exposed to water from L1 died.

### Neurotoxicity Signs and Behavior Alterations

Snails treated with water from L1 and TW did not show neurotoxicity signs or behavior alterations. However, 20% and 21% of the snails exposed to water from L2 and L3, respectively, presented abnormally protruding of the head-food region.

### Number of Eggs and Embryonated Eggs

Table 3 shows the number of eggs and embryonated eggs per mass for each treatment. No statistically differences were observed between treatments (ANOVA, *p* > 0.05). Also, no statistically differences were observed between the number of eggs and embryonated eggs (ANOVA, *p* > 0.05).

**TABLE 3:** Number of *Biomphalaria straminea* eggs and embryonated eggs per mass. TW: tap water; L1, L2, and L3: samples sites, Lugano Lake. No significant differences between treatments were observed (ANOVA, *p* > 0.05).

Water samples	Number of eggs per egg mass	Number of embryonated eggs per egg mass
	Mean ± SD	Mean ± SD
TW	11.00 ± 3.16	11.00 ± 3.16
L1	14.58 ± 6.24	14.25 ± 6.30
L2	18.06 ± 5.55	17.94 ± 5.61
L3	14.58 ± 4.06	13.75 ± 4.90

### Egg masses’ Morphological Changes and Development Delays

**FIGURE 2:**
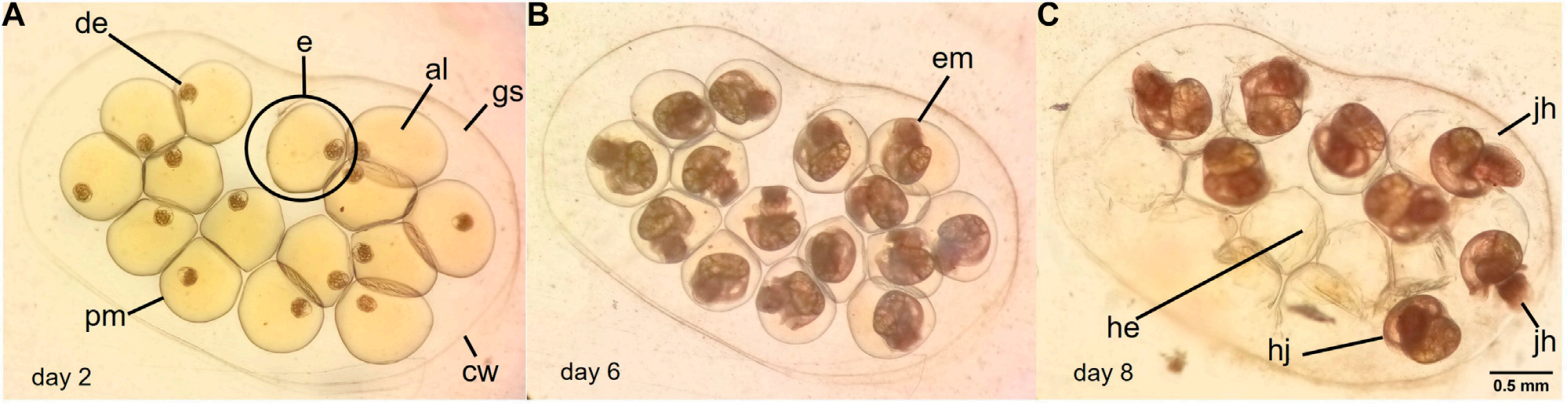
Egg mass with 15 eggs from *Biomphalaria straminea* adult snail treated with TW. The egg mass was in TW too. **(A)** day 2 of development, **(B)** day 6, **(C)** day 8. e: egg, de: developing embryo, al: albumen or perivitelline fluid, pm: perivitelline membrane, cw: capsular wall; gs: gelatinous substance, he: hatched egg, jh: juvenile hatching, hj: hatched juvenile.

Figure 2 shows different photographs of an egg mass (in TW) from *B. straminea* adult snails with TW. Normal development was observed being the time to hatching = 8 days. Morphological alterations were not observed in the eggs or egg masses exposed to TW. Embryos with delayed or arrested development were not observed either (Table 4).

**TABLE 4:** Percentages of *Biomphalaria straminea* egg masses with overlapping eggs, abnormal eggs and arrested eggs. TW: tap water; L1, L2, and L3: sites of sampling, Lugano Lake, Buenos Aires.

Water samples	% Egg masses with overlapping eggs	% Egg masses with abnormal eggs	% Egg masses with arrested eggs
TW	0	0	0
L1	25	25	17
L2	28	17	28
L3	17	8	42

**FIGURE 3:**
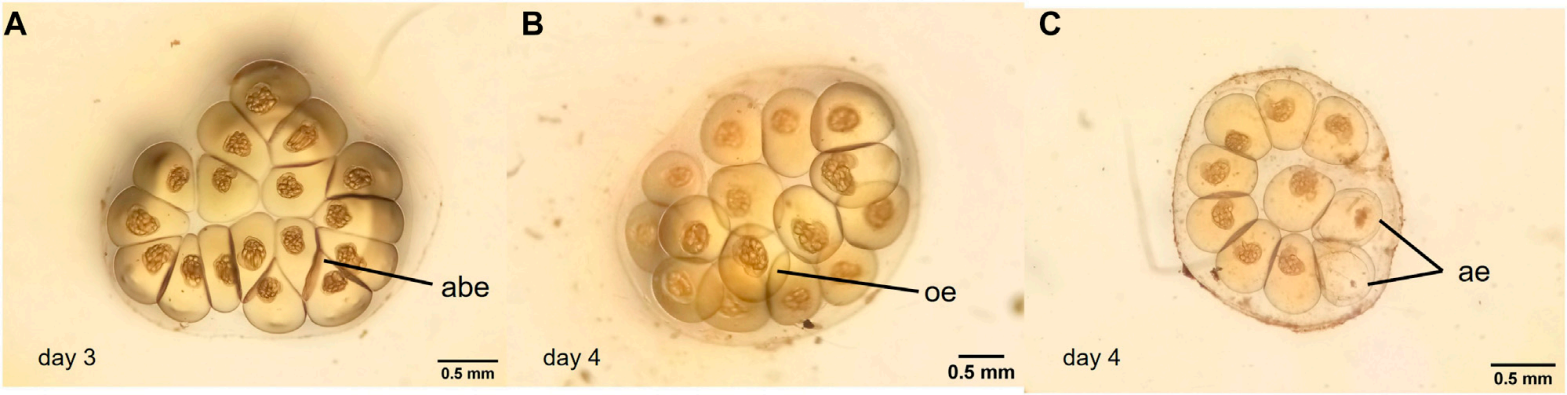
Photographs of abnormal *Biomphalaria straminea* egg masses treated with different water samples from Lugano Lake. **(A)**: egg mass with abnormal eggs, **(B)** egg mass with overlapping eggs, **(C)**: egg mass with arrested eggs. abe: abnormal egg; oe: overlapping eggs; ae: arrested eggs.

On the contrary, some alterations were observed in the eggs and egg masses coming from adult snails treated with water from L1, L2, and L3. Egg masses with some alterations (eggs with morphological changes, overlapping eggs and arrested eggs in different states of development) were observed (Figure 3). Table 4 summarized the percentage of egg masses with each alteration per treatment.

**FIGURE 4:**
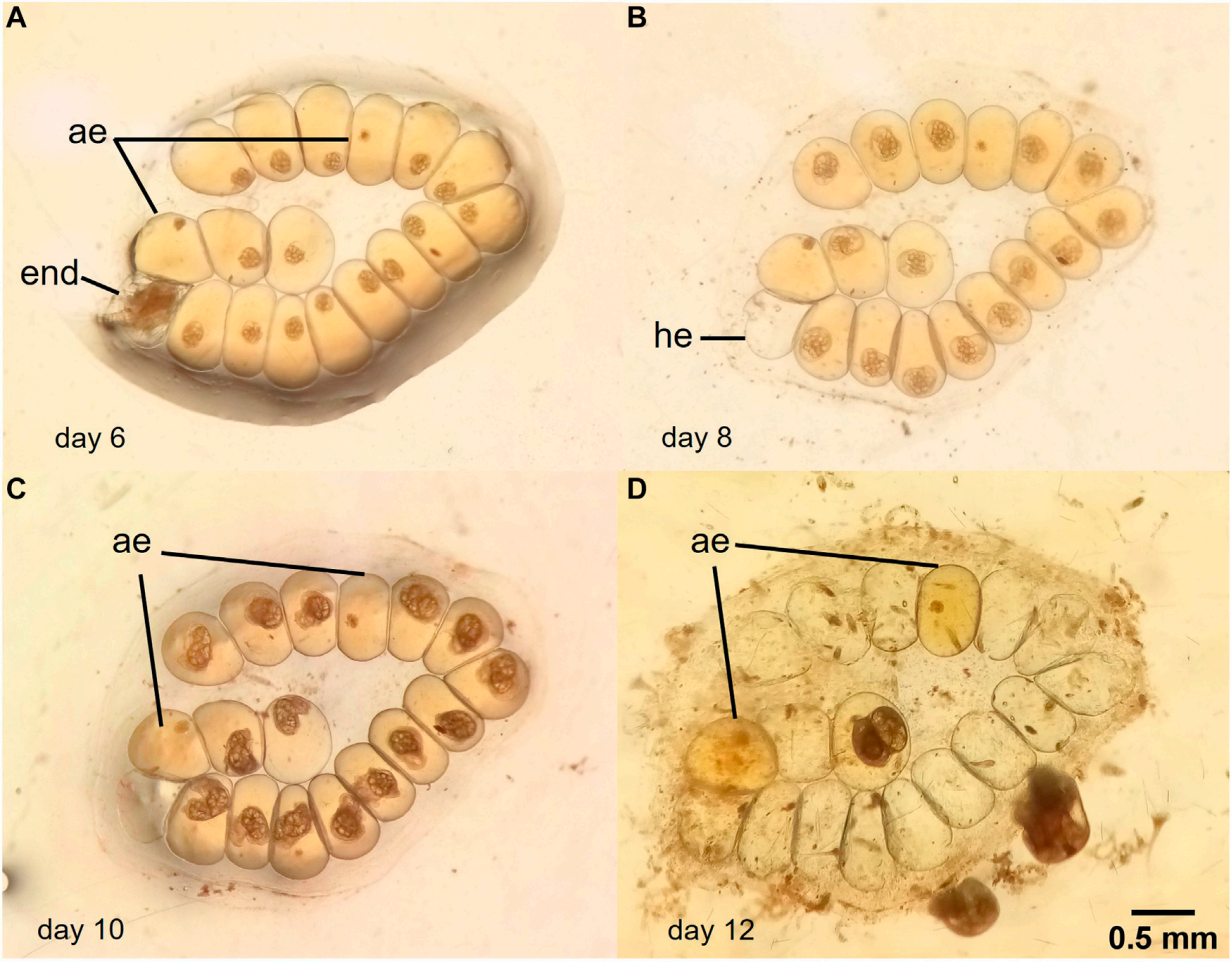
Photographs of an egg mass of *Biomphalaria straminea* at different stages of development of the embryos. The egg mass was exposed to water from site L2, Lugano Lake. **(A)**: day 6 of embryos development, **(B)**: day 8, **(C)**: day 10, **(D)**: day 12. ae: arrested eggs; end: embryo normal development; ae: arrested egg; he: hatched egg.

Also, some egg masses treated with water from the lake (L1, L2, and L3) showed loss of synchronicity in the development. Figure 4 shows a mass with 18 eggs exposed to water from L2. 1 embryo presented normal development and hatched at day 8, 2 eggs showed arrested development in early stages and 15 embryos had delayed development and hatched at day 12.

### Hatching Rate

**FIGURE 5:**
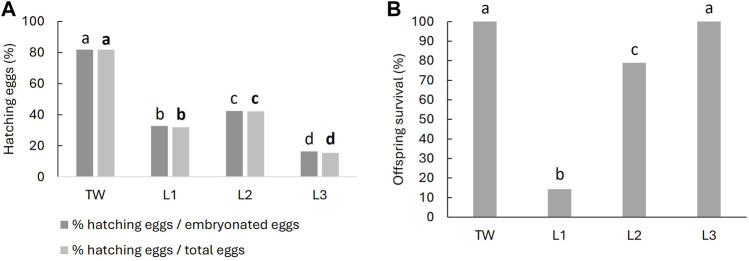
**(A)**: Percentage of hatching eggs and **(B)**: Percentage of offspring survival at the first month after the start of the bioassay in *Biomphalaria straminea*. Egg masses were treated with water from Lugano Lake (L1, L2, and L3 sites) until hatching. After hatching, juveniles were transferred to tap water (TW). Also, egg masses in TW were transferred, after hatching, to clean TW. Statistical differences between sites are indicated with different letters (Chi-squared test, *p* < 0.05).

Statistically differences between treatments were observed in the % of hatching expressed on the number of eggs and on the number of embryonated eggs (Chi-squared test, *p* ˂ 0.05). Egg masses in TW showed 82% of hatching, in water from L1 33%, from L2 42% and the lowest hatching rate was registered in the egg masses treated with water from L3 (16%–15%) (Figure 5A).

### Juveniles Survival at the First Month

After hatching, juveniles were transferred to TW in the same well and the number of alive juveniles were counted after 1 month. The total of snails treated with TW and water from L3 continued alive after the first month. On contrary, a significantly lethality (Chi-squared test, *p* ˂ 0.05) was observed in snails exposed to water from L1 (86%) and L2 (22%) (Figure 5B).

### Juveniles Survival After the Second, Third and Fourth Months

During the second month, 40% of lethality was observed in the group exposed to water from L1. On the contrary, only one snail died in L2 and L3 groups after 2 months. Mortality did not increase in the following months. In regards to snails treated with TW, only one snail died after the third month.

## Discussion

The ecological Reserves of Buenos Aires City are green spaces that improve the life quality of people directly and indirectly by favoring the access to recreation spaces and the protection of the species. The quality of the recreational water in urban green areas generated an impact on soil, vegetables, aquatic and terrestrial life and public health (López Sardi and Larroudé, 2020). In particular, the Lago Lugano Ecological Reserve is located in a basin that receives industrial effluents and domestic discharges. In the last few years, different strategies have been implemented to reduce its contamination, such as phyto and bioremediation techniques. In order to assess water quality, different physicochemical parameters, metals and fecal coliforms have been determined in the lake (Allende et al., 2019; López Sardi and Larroude, 2020). However, we emphasize the importance of carrying out ecotoxicological evaluations, which allow for a more complete outlook in water monitoring and environmental risk assessment (Burgess et al., 2013). Allende et al. (2019) have reported the use of phytoplankton functional group classifications as a tool for biomonitoring the lake. This work reports, for the first time, toxic effects of water contamination on a native species, mainly on the early development stages and provides different endpoints as sensitive biomarkers of pollution.

Conductivity reflects the concentrations of macro-ions. The dissolved nutrients are assumed to increase proportionately with increases in total ions, so this parameter may serve as a primary indicator of total nutrients. Several authors point out that the conductivity is one of the variables that best discriminate the water quality between the sample points with different degrees of anthropogenic impact. Different compounds discharged into surface water such as household products, industrial and agricultural wastes can alter the content of ions and dissolved solids, affecting conductivity (USEPA, 2000; Primavesi, 2002). The results of this work show similar conductivity levels between the three sampling sites of Lugano Lake (1,430 ± 32 μS cm^−1^) that were much higher than those determined for TW (270 μS cm^−1^). In agreement, Allende et al. (2019) reported similar conductivity values in this urban lake in concordance with high levels of nutrients. Also, these authors determined similar values of DO. Adverse ecological effects associated with nutrient enrichment include reductions in DO and the occurrence of harmful algal blooms. The decrease in water clarity (increase in turbidity) can cause loss of macrophytes and the occurrence of dense algal mats that may reduce habitat availability for aquatic organisms. Thus, nutrient enrichment may alter the native composition and species diversity of aquatic communities (USEPA, 2000). Moreover, the inorganic nitrogen compounds may be indicators of contamination from domestic sewage and municipal effluents. Argentina National Legislation (Argentina Hazardous Waste Law N^º^ 24-051, 1993) established the maximum permissible level of different parameters in the guidelines for the protection of freshwater aquatic life in surface waters. Ammonium concentrations were found to be above these guideline levels at the sampling sites. The mean values for the Lugano Lake samples was 4.2 ± 0.5 mg NH₄⁺ L^−1^, while the guidelines established was 1.37 mg NH₄⁺ L^−1^. In addition, water from L2 also showed high nitrite contents, exceeding guideline levels established for this compound (0.06 mg NO₂^−^ L^−1^) by more than ten times. Moreover, high phosphate (PO_4_^3‒^) concentrations were also determined (around 1 mg L^−1^ as PO_4_^3−^ -P), and these values even exceeded the limits established by Res.283/19 (ACUMAR (Autoridad de Cuenca Matanza Riachuelo), 2019) for total phosphorus (TP) concentration (0.01 mg L^−1^) (ACUMAR (Autoridad de Cuenca Matanza Riachuelo), 2019). High DO levels were previously observed in association with algal blooms. Allende et al. (2019) described strong fluctuations in the DO levels throughout the annual cycle, showing concentrations as low as 0.21 mg L^−1^ up to supersaturation values (20.65 mg L^−1^). These authors detected the presence of potentially toxic filamentous Cyanobacteria, as well as bloom episodes and confirmed the eutrophic/hypertrophic conditions of the Lugano Lake due to the values of transparency, nutrients and chlorophyll α concentrations.

Yipp (1983) described that *B. straminea* is a species able to survive within a wide variety of habitats and environmental conditions such as hard water, warm temperatures and eutrophic habitats. In concordance, the exposure (7 days) to water samples from Lugano Lake did not cause significant lethality in this species. Previously, we have reported the same response in these organisms exposed for 14 days to the pesticides azinphos-methyl, carbaryl and acetamiprid. However, sub-lethal effects including variation of antioxidant defenses, ROS and detoxifying enzymes were observed (Cossi, 2019; Cossi et al., 2018, 2020). The use of biomarkers is a useful strategy to assess water contamination and risk to species. In this context, gradual responses, such as biochemical biomarkers, provide less conclusive results on the state of the environment. On the other hand, quantal variables (that occur or not) are relevant endpoints for environmental biomonitoring. For example, effects on behavior, morphology and success on reproduction directly indicate that there are unfavorable conditions that generate negative effects at individual and at higher levels of organization.

Some studies correlated the entire head-foot region visible out of the shell with neurotoxicity and severe toxic effects in the freshwater snail *Chilina gibbosa* (Bianco et al., 2013; Herbert et al., 2021). *B. straminea* exposures in laboratory conditions to neurotoxic pesticides did not show neurotoxicity signs (Cossi et al., 2018; Cossi, 2019). However, 20% of the snails exposed to water from L2 and L3 presented abnormal protrusion of the head-foot out of the shell. In future works we will try to elucidate whether this effect could be related to pollutants present in the lake, mainly to neurotoxins from algal blooms.

Environmental pollutants may have negative consequences in the reproduction success, affecting the species survival at a local scale. In addition, the adverse impact in mollusk populations may negatively affect macroinvertebrate and vertebrate species through the food web, affecting the total ecosystem. *Biomphalaria* spp. have been indicated as useful models for assessing chemical toxicity using early development stages mainly during the embryogenesis process (Caixeta et al., 2022).

Embryos malformations, arrested eggs and decrease in the hatching rate were reported in *B. glabrata, B. alexandrina* and *B. pfeifferi* exposed to chemical compounds (Oliveira-Filho et al., 2005; Adenusi and Odaibo, 2009; Kristoff et al., 2011; Omobhude et al., 2017; Abdel-Tawab et al., 2021; Caixeta et al., 2021). However, none of these responses were observed in *B. straminea* exposed to pesticides in laboratory conditions (Cossi, 2019; Cossi et al., 2018, 2020). Authors described that due to embryos are surrounded by different membranes and substances they were protected against the pesticide. In concordance, low bioavailability and toxicity of silver nanoparticles (Araújo et al., 2020) and ivermectin (Katz et al., 2017) to *B. glabrata* embryos were associated with the protective role of the gelatinous membrane (Caixeta et al., 2022). *B. straminea* snails exposed to water from Lugano Lake laid egg masses with morphological changes including eggs with abnormal forms such as non-round eggs, overlapping eggs and eggs with arrested development at different stages. Also, loss in the synchronicity of hatching and decrease in the hatching rate were observed. Hatching inhibition can be related to developmental delays, interactions with gelatinous membrane and enzyme activities inhibition (Caixeta et al., 2022). Our results could indicate that one or a mix of compounds present in the water samples have high permeability, reached the embryos and caused negative effects on development and hatching. However, it is still not clear whether the permeability of the membranes changes the uptake and bioaccumulation depending on the type of compound (Caixeta et al., 2022).

Even more, morphological changes, arrested eggs and embryos’ lethality determined in this work, could also be a consequence of the exposure of adult snails. Both adult and egg masses were exposed to water from the lake, therefore, in order to be able to discern between them, the exposure of adults and egg masses would also have to be carried out separately.

Survival of hatched juveniles in *Biomphalaria* spp exposed to chemical compounds was less studied. In *B. straminea* exposed to water from L1 and L2, the survival of juveniles significantly decreased after the first month, despite being transferred to TW after hatching. This design was carried out because the nature of the contaminants or the microorganisms present in the water was not known. Therefore after 4 months it is expected that a physicochemical and biological degradation process occurs. On the other hand, the largest discharges take place mainly after rains and floods and this design allows the evaluation of the recovery process of juveniles after exposure. *B. straminea* juveniles exposed 1 month to azinphos-methyl showed high percentage of mortality and, considering that the embryos inside the egg mass had a normal development and hatch, authors described that lethality could had be due to the direct contact with the insecticide (Cossi et al., 2018). Therefore, offspring lethality could have been higher if the juveniles of *B. straminea* had been exposed to the water samples from Lugano Lake. The lowest percentage of hatching was registered in embryos exposed to L2. However, juveniles from L2 survived after the first month indicating that abnormal embryos died inside the eggs. The highest lethality of the juveniles after the first and second months was observed in the group treated with water from L1, site that could receive water from Cildañez Stream and Riachuelo River. The results indicate that, depending on the site, the snails die in the process of embryogenesis or after hatching while tolerant juveniles kept in water can survive.

Long-time exposures would imply a risk for this species in Lugano Lake. Taking into account the chemicals that have been reported in the lake, heavy metals might be one of the causes of the adverse impact observed on reproduction. Decreases in hatching and juvenile survival were observed in *B. glabrata* exposed to Cd, As and Pb (Salice and Roesijadi, 2002; Ansaldo et al., 2009). In the future, we project to measure some organic and inorganic contaminants to provide data of water quality and to be able to relate them with the toxic effects found in this species.

At a local scale, our results become relevant due to the scarce use of invertebrates for water biomonitoring and the need to include invertebrate species as indicators of environmental health in management and government decisions. On the other hand, these assessments are necessary to develop standardized methodologies with native species. In this sense, although the use of globally standardized species has many advantages (Newman, 2014), the native species generate results that can be better extrapolated to the biomonitoring environment. Snails and egg masses in control conditions (TW) showed normal reproduction parameters (time to hatching, number of eggs and embryonated eggs, normal developmental, hatching rate) and offspring survival being similar to those obtained previously in the laboratory in the same conditions (temperature, pH, semi-static conditions, type of exposure containers and control water) (Cossi et al., 2018). These results indicate that it is a reproducible test, a necessary condition for the standardization of the method.

Our results also provide useful tools to improve gastropod representations in global ecotoxicological risk assessment. In this sense, the Organization for Economic Co-operation and Development (OECD, 2010) has recognized the lack of guidelines for the evaluation of the toxicity of chemical substances on the life cycle of mollusks, considering them as species ecologically relevant.

Finally, we highlight the relevance of reproduction assays for assessing chemical toxicity in laboratory conditions and environmental samples. In this way, *Biomphalaria* embryotoxicity test has been recently proposed as a suitable approach for toxicity assay of traditional and emerging pollutants and environmental risk assessment (Caixeta et al., 2022). However its last use is still less studied (Tallarico et al., 2014; Siqueira et al., 2021) indicating that further information is necessary for developing global standardized test protocols. In this sense, our results provide the first assessment of environmental samples on the reproduction of *B. straminea* including oviposition, embryotoxicity and offspring survival.

## Conclusion

Despite the fact that the Lugano Ecological Reserve is a protected area, water samples from different sites generate negative effects on the reproduction, development and juvenile survival of *B. straminea*. Arrested development and the decrease in hatching success and offspring survival were the most important effects caused by water pollution. Water from L1, which could directly receive water from Cildañez Stream and Riachuelo River caused the highest negative impact in this species. The high toxicity observed in *B. straminea* suggests the presence of pollutants in Lugano Lake, that is consistent with the high values of some physicochemical parameters and the presence of high algae densities. *B. straminea* results a sensitive local species to water contamination that could be incorporated in environmental management. *Biomphalaria* spp. reproduction assays can provide a valuable endpoint for toxicity and risk assessments and a usefulness tool for biomonitoring water quality.

## Data Availability

The raw data supporting the conclusion of this article will be made available by the authors, without undue reservation.
